# The Power Law Characteristics of Stock Price Jump Intervals: An Empirical and Computational Experimental Study

**DOI:** 10.3390/e20040304

**Published:** 2018-04-21

**Authors:** Hongduo Cao, Hui Ouyang, Ying Li, Xiaobin Li, Ye Chen

**Affiliations:** 1 Sun Yat-sen Business School, Sun Yat-sen University, Guangzhou 510275, China; 2 Cheung Kong Graduate School of Business, Beijing 100738, China

**Keywords:** stock price jump intervals, jump-diffusion model, power law, computational finance experiment, artificial stock market

## Abstract

For the first time, the power law characteristics of stock price jump intervals have been empirically found generally in stock markets. The classical jump-diffusion model is described as the jump-diffusion model with power law (JDMPL). An artificial stock market (ASM) is designed in which an agent’s investment strategies, risk appetite, learning ability, adaptability, and dynamic changes are considered to create a dynamically changing environment. An analysis of these data packets from the ASM simulation indicates that, with the learning mechanism, the ASM reflects the kurtosis, fat-tailed distribution characteristics commonly observed in real markets. Data packets obtained from simulating the ASM for 5010 periods are incorporated into a regression analysis. Analysis results indicate that the JDMPL effectively characterizes the stock price jumps in the market. The results also support the hypothesis that the time interval of stock price jumps is consistent with the power law and indicate that the diversity and dynamic changes of agents’ investment strategies are the reasons for the discontinuity in the changes of stock prices.

## 1. Introduction

The jump-diffusion model (Merton, 1976) combines the continuous sample path and the stochastic jump process together with the “abnormal” vibrations described by a “Poisson driven” process [1]. Merton’s model has a concise form and clear logic and has become the standard model in analyzing the discontinuous change of the underlying asset price since it was presented. A “Poisson driven” process of the jump component means the probability of having a jump is the same at any time. The follow-up studies generally focused on distribution of jump sizes. Kou’s asymmetric double exponential jump diffusion model has the greatest effect; however, it is obvious that the Poisson process cannot describe such phenomena as the volatility clustering together in an actual financial market [2].

Lots of studies have showed that human activities yield power-law in many fields such as urban population [3], firm size [4], and social networks [5]. Some researchers unravel the dynamic mechanism of power-law based on entropy [6,7]. Focusing on human behavior, some queuing process-based decision-making can originate from the burst and fat tail in human dynamics (Barabási, 2005) [8]. Paying attention to the feature of human behavior, some models are put forward based on human interest, memory or social interaction (Alexei, 2007a, 2007b) [9,10]. Because many individual investors’ (or institutes’) decision-making activities are a deciding factor of the movement of asset prices, the characteristics of asset price volatility should be consistent with the general statistical law of human activities. Considering human dynamics, the jump diffusion model of power law (JDMPL) is given out (Cao Hong-duo et al., 2011) [11], which can depict the sharp kurtosis, fat tail volatility clustering fairly well. An empirical study based on JDMPL shows that the Fokker–Planck distribution is more suitable for describing the distribution of stock jump intervals rather than the traditional exponential distribution on HengSheng Index (HSI) [12].

Farmer and Foley mentioned that agent-based modelling was a better way to help guide financial policies [13]. In order to explain the behavior mechanism of jump intervals, we established an artificial stock market (ASM) and adopted a computational experiment approach to perform financial simulations. The popularity of behavioral finance has increased considerably since its introduction in the 1980s, and the real-world hypotheses and explanations to market anomalies derived from this branch of finance have gained wide recognition [14,15,16,17,18,19,20]. The exclusion of the “agent homogeneity and rationality” presuppositions renders it extremely difficult to determine analytical solutions using mathematical modeling, primarily because establishing a model for every type of agent in the market is arduous and these models cannot reflect overall behavior. The agent-based computational finance (ACF) provides new methodologies and approaches for the research of financial markets. Compared with traditional financial economics research, this branch of finance neither advocates the passive observation of data (empirical approaches) nor relies on mathematical models (logic approaches). Rather, it uses specific “experiment” approaches to determine the underlying regularities of financial phenomena [21]. ACF is ideal for imitating the complex adaptive systems (CAS) in financial markets (Holland, 1992) [22].

The paper is organized as follows. In Section 2, we recapitulate agent modeling and ASM in ACF. In Section 3, we establish an ASF and then the ASM is simulated to observe the overall performance of the market and compare the results with the real world, which is done to determine the distribution conditions of the rate of return in the market and relevant influence factors. In Section 4, we empirically research the distribution of Shanghai Stocks Exchange Composite Index (SHCI) and S&P 500 Index’s jump intervals and use the simulation data packets of the ASM to verify and describe the jump-diffusion model with power law (JDMPL) so that the microstructural interpretations are finally proposed to explain the JDMPL. In Section 5, we discuss our results.

## 2. Agent-Based Computational Finance Method

ACF characterizes financial markets to be complex systems containing numerous adaptive heterogeneous agents. It adopts intelligent information technologies to establish models of these agents in existing market microstructures. These models include investment decision behaviors, learning adaptability behaviors, and between-agent interactions. These models are then incorporated into the design of a series of trading mechanisms and market environments to establish various financial markets, such as stock markets. Finally, the changes in the microstructures of the markets are simulated to elucidate the dynamic characteristics of the markets and their causes of these characteristics.

ACF adopts a bottom-up modeling approach to characterize each agent type in a market and verify the heterogeneity of the agents in the market. Coincidently, the concepts of ACF are consistent with the assumptions in financial economic theories proposed in the 1980s or later. The introduction of ACF shifted early financial research, which is predominantly based on canonical and empirical analysis approaches, to scientific experimentation, improving the scientific value and controllability of financial research. ACF applications reflect the nonlinear mechanisms between internal agents and the emergent properties resulting from these mechanisms.

The ideologies of agent-based ACF research comprise establishing a conceptual model, designing the constructs of an artificial financial system, developing the artificial financial system, operating the artificial finance market (AFM), and explaining and innovating financial theories. Agent modeling and ASM design are the key factors of ACF.

### 2.1. Agent Modeling

ACF emphasizes the heterogeneity, learning capacity, and evolution capability in agent design. The bottom-up modeling approach of computational experimentation facilitates modeling of heterogeneous agents. Nonetheless, agents in the market are vastly different. A theory that accurately determines the number of agent types within a market is nonexistent. Moreover, independently modeling every agent type within a market is impossible because of technical limitations. Thus, the effective classification of agents and appropriate characterization of agent types are extremely crucial to a successful artificial market. Some review of the literature has shown that the majority of previous scholars are inclined to characterize agents based on a single attribute that facilitated their research objectives and then determine whether the particular attribute considerably influenced the research issue.

In behavioral finance, which provides a theoretical basis for ACF, agents’ psychological behaviors, such as cognitive bias, are obtained from real markets. According to previous studies on behavioral finance, discrepancies exist between agents’ information processing ability, cognitive structures, risk aversion attitudes, and decision rules. These aspects can be used to categorize agents.

Generally, ACF is the process of designing and operating an AFM, which inevitably involves the participation of agents. Therefore, determining agents’ decision-making processes is essential. In this context, a number of studies directly categorized agents based on their trading strategies, disregarding other basic attributes and characteristics. This categorization approach eliminates the requirement to process agents’ other independent characteristics and focuses on the influences that the changes in strategy or other market mechanisms have on macrostructure of the overall market.

Numerous studies concerning the categorization of agents’ investment strategies are available. Studies largely categorized agents into fundamental and technical traders to determine the influence that the interactions between the two types of agents have on price. For example, Kirman and Teyssie (2001) categorized traders in the market into fundamentalists and chartists (noise traders) [23]. Gao et al. (2005) categorized traders into fundamental and technical traders, where fundamental traders base their investments on anticipated basic stock value and technical traders base their investments on trading history [24]. Subsequent studies extended beyond this categorization method. Based on traditional finance theories (rational expectations), behavioral finance theories, and noise trader theory, agents can be categorized into four types based on their investment strategies: specifically, rational expectation strategies, Barberis–Shleifer–Vishny (BSV) strategy, noise trader strategy, and passive trading strategy [25].

In addition to directly categorizing the investment strategies adopted by agents, studies have also categorized agents using intrinsic factors to determine which factors influence the trading strategies adopted by agents and categorize agent types. For example, Zhang and Zou (2011) categorized agents into those that make risk decisions based on traditional expected utility theory and those that make decisions based on prospect theory to determine whether investment strategies based on traditional decision-making preferences or those based on prospect theory are more favorable for agents [26]. Kyle et al. (1984, 1989) categorized participating agents into three types: namely, noise traders, or traders who lack the ability to forecast stock prices or make stochastic stock forecasts; uninformed traders, or retail traders that lack insider information or make stock forecasts based on previous prices or estimated dividends; and informed traders, or traders who receive dividend distribution information or possess the ability to accurately estimate potential value [27,28]. Klugera and McBride (2011) categorized agents into informed and uninformed (liquidity-motivated) agents. Both types of agents are capable of learning to trade but are zero-intelligence on all other behaviors [29]. Other studies have investigated the influences that the execution methods of trading strategies have on the market, in which agents are categorized based on their level of patience to elucidate the influences that agents’ response times to market messages have on the market. Foucault, Kadan, and Kandel (2005) categorized agents into patient and impatient traders. Ma et al. (2011) expanded the categorization of agents into patient, impatient, and lenient traders. This categorization is used to determine the influence that an increase in patient traders in the market has on market depth, bid-ask spread, and order turnover rate [30,31].

In addition to satisfactory agent categorization and modeling, ACF also takes into account that agents are capable of learning and evolving. Learning refers to agents’ ability to summarize or reflect on their own or others’ past trading experiences, remember and improve on successful trading experiences, and discard failed trading strategies to increase investment return and probability of success. Evolution refers to the withdrawal of failed agents from the market and the addition of new agents into the market over time. These changes cause the overall market to fluctuate and evolve in the long term. Thus, ACF’s agent evolution is consistent with real market conditions.

The earliest agent learning mechanism is introduced in the ASM developed by the Sante Fe Institute (SFI-ASM). In the SFI-ASM, agents possess an *m*-th number of rules. These rules are placed into specific orders for different situations based on their individual strengths. Stronger rules are placed closer to the beginning of the order. After each trading behavior, the agent adjusts the strength of each rule and reorders them. Agents can further apply GA to update these rules, generate new rules through mutation and crossover, and replace old mechanisms with new ones. Some have expanded agent independent learning into social learning, where agents not only learn from personal trading experience but also learn from surrounding agents and the market. Chakrabarti and Roll (1999) modeled a market composed of agents who combine their own private information with rational learning about the information possessed by others and compared this market with a market populated by agents who receive the same private information but ignore other agents [32]. Hu and Weliman (2003) developed a dynamic multiagent system, in which the behaviors of individual agents are dependent on the behaviors of other agents [33]. Chen and Yeh (2001) developed the Artificial Intelligence Economic Research Center Artificial Stock Market (AIE-ASM) using genetic programming. The researchers incorporated social learning behaviors into ASM and adopted a “business school” approach to distribute rule messages vertically from one agent generation to the next [34]. Other scholars have combined two or more approaches. For example, Kendall (2003) developed an artificial market in which agents are capable of both independent and social learning [35].

### 2.2. AFM Designs

AFM designs comprise subjects (agents), objects (tradable asset types and volume), information formation and transmission methods, market trading mechanism designs, and relevant rules (e.g., price limit).

A number of market models included banks and government departments as the market subjects. However, the purpose of the present study is to examine the influences that agents’ microstructural behaviors have on the macrostructural characteristics of the market. Therefore, we modeled general agents as the participating subjects. Previous studies have adopted institution agents as the subjects and other departments as the external environment in their models to account for influences of institutions. This approach simplified the research issue.

Tradable securities, the object in AFMs, are an essential component of computational finance experiments. In agent-based models, the types of tradable securities are typically limited. The modeling of various agent types and the design of learning mechanisms render the entire experiment extremely complicated. Hence, the design of securities types is generally simplified. The most common approach for designing securities types is to assume that the market comprises two types of assets, risk assets and risk-free assets (assets without risk generate fixed, risk-free revenue) [36]. Cincotti (2003) extended the number of investment products in modeling an ASM containing numerous stocks to analyze its times series characteristics [37]. In actuality, a single stock that reflects the market index can be incorporated. This singular stock represents the entire stock market. Then, risk-free assets can be added to achieve efficient market coverage and simplify the experience process.

A number of scholars have proposed methods to process market information based on the aforementioned market subjects and trading targets. Market information includes the dividend and earnings of securities and other basic factors, as well as the positive and negative messages that influence financial asset prices. Incorporating market information diversifies ASMs and enhances their conformity with real markets. However, previous studies have rarely focused on the market information in ASMs because of the unpredictability in the method and the frequency at which such information is presented.

The price formation mechanisms of AFM can largely be categorized into three types: namely, market maker mechanisms, dealmaker mechanisms, and trading mechanisms that imitate the market (e.g., call and continuous auction mechanisms).

Market maker mechanisms use the difference between supply and demand to adjust prices. Under these circumstances, the market never achieves true equilibrium. Early studies largely adopted market maker mechanisms to model their artificial markets. When market maker mechanisms are adopted, the market maker sets a price. Agents submit their buy and sell orders based on their own circumstances. All buy and sell orders are then collated. Stock prices rise when demand surpasses supply and fall when supply surpasses demand. Driven by market maker mechanisms, price changes concurrently and proportionally with oversupply.

Dealmaker mechanisms assume that certain demand functions exist in the market and recalculate current market clearing prices following a specific period interval. Brock and Hommes (1997) adopted a dealmaker mechanism to verify trading prices. Specifically, they specified that total demand equals total supply, which is an equilibrium price principle proposed in microeconomics [38]. Compared to market maker mechanisms, the inventory of market makers is not an issue for price formation mechanisms because the clearing price is recalculated after a certain period. However, price discontinuity is likely to occur when using price formation mechanisms.

The aforementioned studies show that both market maker and dealmaker mechanisms adopt mathematical modeling and analytical solutions to determine trading prices. These price determination methods are supported in theory by various studies. However, they are sometimes different from the price formation methods used in real markets (e.g., price formation mechanism adopted in the Securities Market of China), suggesting that loopholes concerning price formation mechanisms exist in artificial market designs. In response, a number of scholars have applied real-world trading mechanisms (e.g., order-driven mechanisms) into ASM, such as the call auction mechanism, the continuous auction mechanism, or the combined call-continuous auction mechanism, in the attempt to create an artificial market that closely reflects real markets. In artificial markets based on order-driven mechanisms, agents submit their buy and sell orders. Buy and sell orders are then matched using the trading regulations of real stock markets to form trading prices.

Each of the aforementioned three methods has its own strengths and weaknesses. The first two methods produce prices that closely reflect theoretical values. They are also easier to implement, but an equilibrium solution is required. The order-driven mechanism can better reflect the specific conditions of the real market. It is more suitable for market simulations, but it is also comparatively more difficult to implement than the first two methods. A review of current trends shows that the application of the order-driven mechanism in studies concerning China’s stock market is becoming increasingly popular. In the present study, we modeled the ASM based on a combined call-continuous price formation mechanism.

In addition to the aforementioned trading subjects, objects, trading mechanisms, and market information, a number of scholars have incorporated additional market constraints and conditions.

## 3. Artificial Market Construction

A novel ASM is modeled in the present study. First, the agents are categorized and described, and the price forecasting and utility function models for the various agent types are established. Second, the trading mechanism is designed, including a continuous auction mechanism and a call auction mechanism. Third, other key parameters and hypotheses for the ASM are formulated. Finally, the overall architecture for the ASM is created.

### 3.1. Agent Modeling

#### 3.1.1. Agent Categorization

Agents adopt different trading strategies in the market. A summary of existing literature and market experiences indicates that the three widely accepted trading strategies employed in China’s stock market are value investing, trend investing, and stochastic investing. These strategies are the top-down approach. The bottom-up categorization method examines the basic attributes influencing agents’ behavioral decisions, such as gender (women/men), age (old/young), and wealth (rich/poor). Further observation of these basic attributes shows that they influence agents’ risk appetite, which consequently affects their investment behaviors. In identical situations, women are less tolerant to risk than men, rich people are less tolerant to risk than poor people, and older people are less tolerant to risk than younger people. Based on these basic attributes, agents can be categorized into eight types: specifically, poor young men, poor old men, rich young men, rich old men, poor young women, poor old women, rich young women, and rich old women. Moreover, an extra type is included in the present study, institution agents. Therefore, agents are categorized into nine types using the bottom-up approach. By combining the two approaches, the nine types of agents each adopt one of three investment strategies, creating 27 agent types.

Let A, B, C, D, E, F, G, H, and I represent poor young men, poor old men, rich young men, rich old men, poor young women, poor old women, rich young women, and rich old women, and institute agent, respectively. Let *k* = 1, 2, and 3 represent value investment strategy, trend investment strategy, and stochastic investment strategy, respectively. The market comprises a total of 108 agents. The number of agents in each category is tabulated in Table A1 of Appendix B.

#### 3.1.2. Basis of Agent Decision Formulation

**Figure 1:**
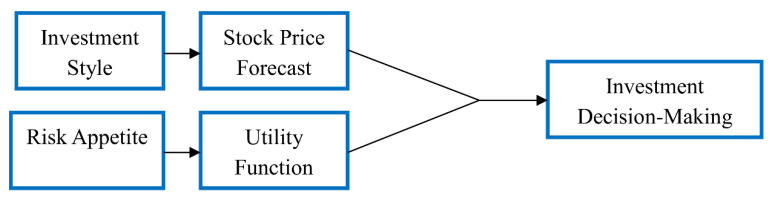
Decision logic.

Agents are categorized based on their investment style and risk appetite. Agents’ investment style influences their forecasts of future stock prices. Agents’ risk appetite directly influences their utility function, and their utility function influences their allocation of risk assets. The two factors collectively influence agents’ investment decision behaviors. The influences that agents’ investment styles have on their price forecasting behaviors are first discussed shown as Figure 1. Then, the influences that agents’ risk appetite has on their asset allocation behaviors are discussed. Finally, how agents combine their investment style and risk appetite to formulate investment decisions is discussed.

(1) Agents’ Investment Strategies

Three types of investment strategies exist in the market: value investment strategy, trend investment strategy, and stochastic investment strategy. The three strategies are evenly adopted by the agents in the market. That is, the three strategies are adopted in all nine agent types to forecast stock prices and formulate investment decisions. The valuation methods for the three strategies are as follows:

(i) Value Investment Strategy

Agents that adopt the value investment strategy believe that the market is efficient and that stock prices fluctuate concurrently with value. They maintain that stock prices constantly trend toward the real stock value. However, agents’ profit expectations vary. Therefore, a stochastic variable is used in the present study to reflect the forecasting differences of different agents, which can be expressed using the following equation:

(1) $${p_{i,t}}^{e}=\frac{1}{30}\sum_{i=1}^{30}p_{t-i}+\delta_{t}$$

where, $p_{i,t}^{e}$ is the stock estimation of value-investing agent *i* in the *t*-th period, $p_{i,t-j}$ is the stock price *j* days ago (*j* = 1, …, 30), $\delta_{t}\in (-2\sigma_{t},2\sigma_{t})$ is the random number that conforms to uniform distribution, and $\sigma_{t}$ is the standard deviation of $p_{t-j}$ (*j* = 1, 2, …, 30). Let ${R^{e}}_{i,t}=\frac{p_{i,t}^{e}}{p_{t-1}}-1$ be the expected rate of return of the value-investing agent in the *t*-th period.

(ii) Trend Investment Strategy

Agents that adopt the trend investment strategy believe that certain trends exist in short-term stock prices. Their investment behaviors are based on historical trends. However, different agents value historical data differently. They believe that recent prices have a greater influence on current stock prices. Hence, they weigh the value of historical data according to time.

(2) $$p_{i,t}{}^{e}=\sum_{j=1}^{30}k_{j}*p_{t-j}+\delta_{i,t}$$

where, $p_{i,t}^{e}$ is the stock estimation of trend investing agent *i* in the *t*-th period, $p_{t-j}$ $p_{i,t-j}$ is the stock price *j* days ago (*j* = 1, …, 30), $\delta_{i,t}\in (-2\sigma_{t},2\sigma_{t})$ is the random number that conforms to uniform distribution, and $\sigma_{t}$ is the standard deviation $p_{t-j}$ (*j* = 1, 2, …, 30). Let $R_{i,t}=\frac{p_{i,t}^{e}}{p_{t-1}}-1$ be the expected rate of return of the trend-investing agent in the *t*-th period.

(iii) Stochastic Investment Strategy

Agents that adopt the stochastic investment strategy are considered “novice” agents. They do not maintain a fixed determination system. Rather, they adopt the opening price in each period as their forecast price.

(3) $$p_{i,t}^{e}=p_{t}^{0}$$

(2) Risk Appetite and Utility Function

Agents’ risk appetite determines their utility function. Consequently, their utility function facilitates them in formulating the optimal asset allocation plan. The proposed market comprises a single risk asset (stocks) and a single risk-free asset (case). Agents own both stocks and cash. Short purchasing and short selling are prohibited.

A novel utility function is modeled based on the research objectives and the utility function model presented in cumulative prospect theory [39] (Bernard and Ghossoub, 2010) to determine the optimal risk asset allocation. In the model proposed by Bernard and Ghossoub, the independent variables for the utility function are the absolute loss values and absolute gain values. These independent variables are appropriate when considering a single agent type because only the monotonicity and marginal diminishment of the utility function must be characterized. By comparison, the utility functions of agents with different financial conditions must be characterized in the proposed model. The initial wealth difference between the agents generates different rates of return in identical gain conditions. Therefore, the absolute gain and absolute loss values adopted in the original model are inadequate for characterizing agents’ different utilities. In the present study, we adopted rate of return as the independent variable for agents’ utility functions. Using rate of return as the independent variable not only accounts for the increase in monotonicity and marginal diminishment of utility functions but also reflects agents’ different financial conditions.

Let $w_{i,t-1}$ be the initial financial condition of agent *i* in period *t*. The wealth of agent *i* comprises a specific amount of stocks (risk asset) and cash (risk-free asset), where $\xi_{i,t-1}$ represents risk asset, ($w_{i,t-1}-\xi_{i,t-1}$) represents cash, and $\xi_{i,t}^{*}$ represents the amount of simulated risk asset (optimal allocation) in the current period.

During the formulation of the optimal asset allocation plan (agent *i*), let the future rate of stock return be $x_{i,t}$, where $x_{i,t}\sim N(\mu_{i,t},\sigma^{2})$. Cash generates risk-free return (r). $y_{i,t}=x_{i,t}-r$ is the expected excess return rate, where $y_{i,t}\sim N(\mu_{i,t}-r,\sigma^{2})$. Let $v_{i,t}=\mu_{i,t}-r$, where $y_{i,t}\sim N(v_{i,t},\sigma^{2})$.

Agents’ expected risk-return can be expressed as follows:

(4) $$D_{i,t}=\xi_{i,t}^{*}*(1+X_{i,t})+(1+r)*(W_{i,t-1}-\xi^{*}{}_{i,t})-(1+r)*W_{i,t-1}=\xi^{*}{}_{i,t}*(X_{i,t}-r)=\xi_{i,t}^{*}*y_{i,t}$$

Hence, the expected risk rate of return is

(5) $$R_{i,t}=\frac{\xi_{i,t}^{*}*y_{i,t}}{W_{i,t-1}}$$

The utility function (*U*) can be expressed as follows:

(6) $$u(R)=\left\{ \begin{array}{l} u^{+}(R)=R^{\alpha }\begin{array}{cc} & R\geq 0 \end{array}\\ -u^{-}(-R)=-\lambda {\left(-R\right)}^{\beta }\begin{array}{cc} & R<0 \end{array} \end{array}\right.$$

The following criteria must be satisfied:

(1) $u^{+}:{\bar{R}}^{+}\to {\bar{R}}^{+},$$u^{-}:{\bar{R}}^{+}\to {\bar{R}}^{+}$, where ${\bar{R}}^{+}=R^{+}\cup \left\{+\infty \right\}$
(2) $u^{+}(+\infty )=u^{-}(+\infty )=+\infty$
(3) 0<α<β,α≤β≤1,λ≥1

The total utility of the agent (V(R)) can be expressed as follows:

(7) $$\begin{array}{l} V_{i,t}(R_{i,t})=\int_{0}^{+\infty }u^{+}(R_{i,t})f(u^{+}(R_{i,t})du^{+}(R_{i,t})+\int_{-\infty }^{0}u^{-}(R_{i,t})f(u^{-}(R_{i,t}))du^{-}(R_{i,t})\\ =\int_{0}^{+\infty }w_{i,t-1}{}^{-\alpha }f_{R_{i,t}}(y)\xi^{\alpha }y^{\alpha }dy-\int_{-\infty }^{0}\lambda w_{i,t-1}{}^{-\beta }f_{R_{i,t}}(y)\xi^{\beta }{\left(-y\right)}^{\beta }dy \end{array}$$

Formula (7) is explained in Appendix A. In this section of the calculation, we excluded the probability of distortion mentioned in prospect theory. To include the probability of distortion, please refer to Zhou et al. (2010) [40]. Let the first-order derivative be 0:

(8) $${V_{i,t}}^{\prime }=\alpha w_{i,t-1}{}^{-\alpha }\xi^{\alpha -1}\int_{0}^{+\infty }f(y_{i,t})y_{i,t}{}^{\alpha }dy_{i,t}-\lambda \beta w_{i,t-1}{}^{-\beta }\xi^{\beta -1}\int_{-\infty }^{0}f(y_{i,t}){\left(-y_{i,t}\right)}^{\beta }dy_{i,t}=0$$

(1) When α=β

If $\frac{\int_{0}^{+\infty }y_{i,t}{}^{\alpha }f(y_{i,t})dy_{i,t}}{\int_{-\infty }^{0}{\left(-y_{i,t}\right)}^{\beta }f(y_{i,t})dy_{i,t}}=\lambda$, then ${V_{i,t}}^{\prime }$ = 0. Maximum agent utility is achieved with any risk asset investment ratio.

If $\frac{\int_{0}^{+\infty }y_{i,t}{}^{\alpha }f(y_{i,t})dy_{i,t}}{\int_{-\infty }^{0}{\left(-y_{i,t}\right)}^{\beta }f(y_{i,t})dy_{i,t}}>\lambda$, then ${V_{i,t}}^{\prime }$ > 0. The agent invests all assets into risk assets.

If $\frac{\int_{0}^{+\infty }y_{i,t}{}^{\alpha }f(y_{i,t})dy_{i,t}}{\int_{-\infty }^{0}{\left(-y_{i,t}\right)}^{\beta }f(y_{i,t})dy_{i,t}}<\lambda$, then ${V_{i,t}}^{\prime }$ < 0. The agent invests all assets into risk-free assets.

(2) When α≠β, let

(9) $$\theta_{i,t}{}^{}=\frac{\xi_{i,t}^{*}}{w_{i,t-1}}={\left(\frac{\alpha }{\lambda \beta }\right)}^{\frac{1}{\beta -\alpha }}{\left(\frac{\int_{0}^{+\infty }y_{i,t}{}^{\alpha }f(y_{i,t})dy_{i,t}}{\int_{-\infty }^{0}y_{i,t}{}^{\beta }f(-y_{i,t})dy_{i,t}}\right)}^{\frac{1}{\beta -\alpha }}$$

and

(10) $$\theta^{*}{}_{i,t}=\left\{ \begin{array}{l} 0\\ \theta^{}{}_{i,t}\\ 1 \end{array}\right.\begin{array}{l} \theta <0\\ 0\leq \theta \leq 1\\ \theta >1 \end{array}$$

Using the aforementioned equations, we obtained the optimal risk allocation proportions in different conditions. We found that the optimal risk asset allocation relies on the α, β, and λ values, where α is the risk aversion coefficient during gain (risk appetite increases concurrently with an increase in α), β is the risk aversion coefficient during loss (risk appetite increases concurrently with an increase in β), λ is the sensitivity of the agent at gain/loss equilibrium (sensitivity increases concurrently with an increase in λ). Therefore, α, β, and λ are closely associated with the three basic attributes (gender, wealth, and age) examined in the present study. In a subsequent experiment, we set different coefficient values for different agent types to obtain different utility functions. The optimal risk asset allocation plan in the current period can be determined based on the different rate of returns forecasted by the agents in each period. The allocation plan served as a basis for the subsequent formulation of decisions.

#### 3.1.3. Agent Decision-Making Methods

Agents formulate trading decisions based on the outcomes of Section 3.1.1 and Section 3.1.2. They rely on two processes to formulate decisions. First, they compare the optimal risk allocation plan with the current risk asset allocation plan ($\xi^{*}{}_{i,t}$ and $\xi_{i,t-1}$). Second, they compare the current stock forecasts with current opening price ($p_{i,t}^{e}$ and $p_{t}^{0}$). Based on the optimal risk asset allocation ($\theta_{i,t}^{*}$) obtained in Section 3.1.2, the optimal risk configuration for the current period can be calculated using the following equation:

(11) $$\xi^{*}{}_{i,t}=\theta_{i,t}{}^{*}*w_{i,t-1}$$

The risk assets in the previous period can be calculated as follows:

(12) $$\xi_{i,t-1}=p_{i,t-1}*q_{i,t-1}$$

where, ($p_{i,b,t},q_{i,b,t}$) is buy orders and ($p_{i,s,t},q_{i,s,t}$) is selling price and volume. The decision process can be expressed as follows:

(1) When $p_{i,t}^{e}\geq p_{t}^{0}$ and $\xi_{i,t}^{*}\geq \xi_{i,t-1}$, agents formulate buy decisions. $p_{i,b,t}\in \left(p_{t}^{0},p_{i,t}^{e}\right)$ is a random variable that conforms to uniform distribution and $q_{i,b,t}=\frac{\xi_{i,t}^{*}-\xi_{i,t-1}}{p_{i,b,t}}$.
(2) When $p_{i,t}^{e}<p_{t}^{0}$ and $\xi_{i,t}^{*}<\xi_{i,t-1}$, agents formulate sell decisions. $p_{i,s,t}\in \left(p^{e}{}_{i,t},p_{t}^{0}\right)$ is a random variable that conforms to uniform distribution and $q_{i,s,t}=\frac{\xi_{i,t-1}-\xi_{i,t}^{*}}{p_{i,s,t}}$.
(3) When $p_{i,t}^{e}<p_{t}^{0}$ and $\xi_{i,t}^{*}\geq \xi_{i,t-1}$, agents formulate buy decisions. $p_{i,b,t}=p^{e}{}_{i,t}$ and $q_{i,b,t}=\frac{\xi_{i,t}{}^{*}-\xi_{i,t-1}{}^{}}{p_{i,b,t}}$
(4) When $p_{i,t}^{e}\geq p_{t}^{0}$ and $\xi_{i,t}^{*}<\xi_{i,t-1}$, agents formulate sell decisions. $p_{i,s,t}=p_{i,t}^{e}$ and $q_{i,s,t}=\frac{\xi_{i,t-1}{}^{}-\xi_{i,t}{}^{*}}{p_{i,s,t}}$.
(5) The aforementioned four conditions can be determined by examining agents’ asset allocation plans (utility function) and price forecasting behaviors. These conditions are then used to define agents’ various decision-making behaviors, ultimately reflecting their limited order patterns. That is, agents submit orders according to ($p_{i,b,t},q_{i,b,t}$) or ($p_{i,s,t},q_{i,s,t}$).

#### 3.1.4. Agent Learning and Evolution

Agents in the proposed ASM are capable of learning and evolving. Agents with learning abilities can collate and reflect personal trading experiences and learn from their own or others’ learning experiences. Agent evolution reflects the evolution of the entire market. Agents that are unsuccessful and lose their wealth are unable to survive in the market and eventually withdraw from the market. These agents are replaced by new agents.

(1) Agents’ Learning Mechanisms

In the proposed ASM, each agent owns a personal account. The trading prices ($p_{i,b,t}^{*}/p_{i,s,t}^{*}$) and trading volume ($q_{i,b,t}^{*}/q_{i,s,t}^{*}$) of every transaction, daily wealth ($w_{i,t}$), and experience account ($N_{i,t}$) are recorded in the personal account. The agents experience account, or his/her trading situation (failure/success), is updated in the personal account every 20 days. A successful transaction is recorded with a + 1, a failed transaction is recorded with a − 1, and an even transaction or no transactions are recorded with a + 0.

A successful or failed transaction is determined by comparing the trading price with the closing price in the same period. The transaction is considered successful if $p_{i,b,t}^{*}<p_{i,t}^{}$ or $p_{i,s,t}^{*}>p_{i,t}^{}$, it is considered a failed transaction if $p_{i,b,t}^{*}>p_{i,t}^{}$ or $p_{i,s,t}^{*}<p_{i,t}^{}$, and it is considered an even transaction if $p_{i,b,t}^{*}=p_{i,t}^{}$ or $p_{i,s,t}^{*}=p_{i,t}^{}$. The *j*th day is set as the “learning day.” First, the agent reviews his/her investment situation in a specific period. That is, the agent compares $w_{i,j}$ and $w_{i,j-20}$ to calculate the number of successful and failed transactions in the specified period. The outcomes are then used for learning and improvement. The rules of this process are as follows:

(i) If $w_{i,j}-w_{i,j-20}\geq 0$, then the agent’s overall investment condition in the specified period is profitable. The agent is satisfied with his/her current investment strategy.
(ii) If $w_{i,j}-w_{i,j-20}<0$, then the agent’s overall investment condition in the specified period is unprofitable. In this instance, the agent further reviews his/her experience account ($N_{i,t}$). If $N_{i,t}<0$, the failed transactions in the specified period outnumber the successful transactions. Hence, the agent changes his/her investment strategy by referencing the strategies adopted by successful agents in the same category. That is, the agent learns from the profit makers ($w_{i,j}-w_{i,j-20}\geq 0$) in the same row of Table 1. When only one profit maker is present in the row, the agent adopts the profit maker as his/her target of learning. When multiple profit makers are present, the system randomly selected one profit maker as the agent’s target of learning. When no profit makers are available, the agent retains his/her investment strategy because he/she considers the failed transaction to be associated with overall market conditions rather than individual strategy.
(iii) If $w_{i,j}-w_{i,j-20}<0$ and $N_{i,t}\geq 0$, then the agent’s successful transactions outnumber failed transactions in his/her trading history. However, the agent remains unprofitable. In this instance, the agent considers the problem to derive from the poor allocation of assets and opts to change his/her utility function (relevant parameters) by referencing profitable agents in the same category. That is, the agent learns from profitable agents ($w_{i,j}-w_{i,j-20}\geq 0$) in the same column of Table 1. When only one profit maker is present in the column, the agent adopts the profit maker as his/her target of learning. When multiple profit makers are present, the system randomly selected one profit maker as the agent’s target of learning. When no profit makers are available, the agent retains his/her investment strategy because he/she considers the failed transaction to be associated with the overall market condition rather than individual strategy.

(2) Agent Evolution

In addition to learning ability, agent renewal and replacement also exist in a normal market. Agents that are unsuccessful eventually withdraw from the market. New agents also enter the market. In the development of the proposed ASM, we assumed that agents enter and withdraw from the market, but that the overall number of agents (*n* = 108) in the market remains unchanged. When $w_{i,t}\leq 5\%w_{i,0}$, agent *i* withdraws from the market and is substituted by a random new agent. The investment strategy and utility function of the new agent are randomly generated.

### 3.2. Trading Mechanism Design

A hybrid call-continuous double auction mechanism is adopted as the trading mechanism for the proposed ASM to maintain consistency with the real market. A continuous double auction mechanism that follows the principle of price/time priority is adopted in the proposed artificial market. Real transactions comprise limit orders and market orders. For limit orders, agents predetermine a buy and sell price. Transactions that are higher than the designated buy price or lower than the designated sell price are not considered. The order is retained for the next transaction opportunity. For market orders, agents are willing to trade at the current market price. That is, they are willing to trade at the price proposed by the counterparty. In the ASM experiment, the orders submitted by the agents are all limit orders. The call auction mechanism adopted by the stock market in China mainland is used in the present study. This mechanism follows the principles of maximum volume, minimum surplus, market pressure, and market reference price. The preceding principle is first executed. If a single price is obtained from the preceding principle, this price is adopted as the final price for the call auction. If multiple prices are obtained, then the other principles are sequentially executed until a single price is obtained.

### 3.3. Other Market Details and Relevant Parameters

In addition to the design of agents and trading mechanisms in the proposed ASM, other factors are set and hypothesized. The proposed market only comprises one type of risk asset and one type of risk-free asset. The rate of return for the risk-free asset is a known value (r) and is cost-free. The initial number of agents in the market is 100. This value remains unchanged throughout the experiment. However, during the simulation, agents may withdraw from the market and be replaced by new agents. The entire simulation comprises 5010 periods. The market parameters are shown as Table 1.

### 3.4. Basic ASM Architecture

#### 3.4.1. Programming Software

The proposed simulation platform for the ASM is developed in C++ using Visual Studio 2008 based on the outlined learning model and learning strategy. The platform fully utilized the three-tier architecture of software design, whereby the platform is divided into a presentation layer, an operating logic layer, and a data access layer to achieve the goals of high internal convergence and low coupling. The platform simultaneously took into account market simulation progress and efficiency.

**Figure 2:**
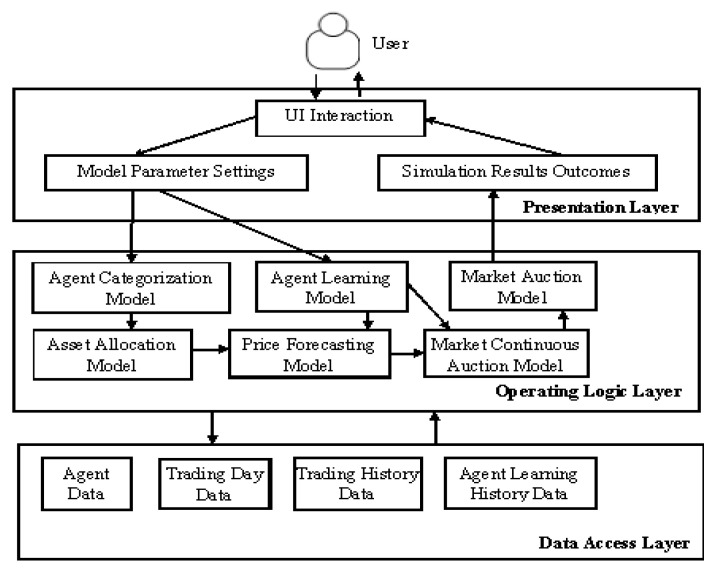
Module composition of the artificial stock market (ASM) simulation platform.

The basic systems and modules of the simulation platform are illustrated in Figure 2. The presentation layer controls the interaction between the user and the platform, including setting model parameters and generating simulation outcomes. The operating logic layer is responsible for the computation and processing of the model and the platform. The data access layer enables the access and archiving of data generated by the platform.

#### 3.4.2. Basic ASM Framework

**Figure 3:**
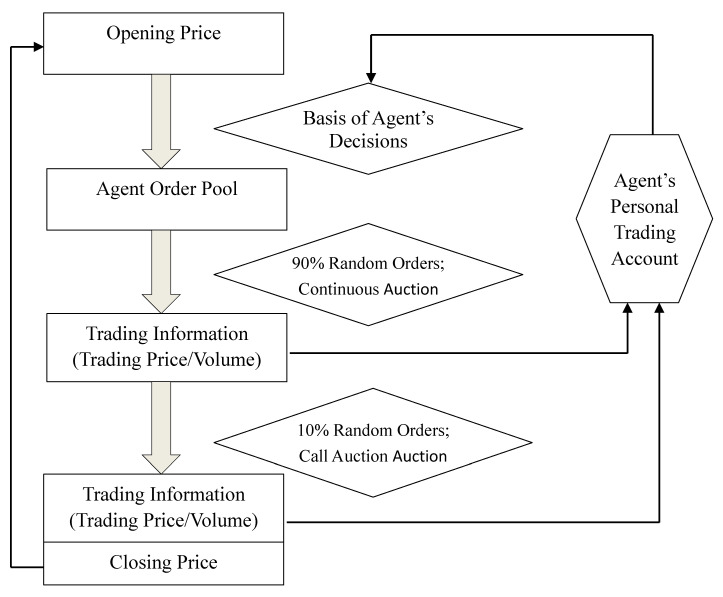
ASM operations.

The detailed operations of the entire market are illustrated in Figure 3.

First, the system randomly generates an opening price in the current period based on a predetermined range of the closing price in the previous period.

Second, agents in the market formulate buy/sell decisions based on the opening price in the current period and their individual price forecasting models and asset allocation models. Their buy/sell orders enter the market and accumulate in an order pool.

Third, orders randomly enter the market throughout the trading day. Among all the orders in the order pool, 90% of the orders undergo continuous auction. The continuous auction trading mechanism is adopted to determine the trading price and trading volume of each order. The trading information is recorded in the corresponding agent’s personal account.

Fourth, 10% of the orders are retained. These orders are added to the unmatched orders in the continuous auction. They undergo call auction to generate the closing price for the current period. The trading volume and trading price are recorded in the corresponding agent’s personal account.

Fifth, agents periodically collate the information in their personal accounts and reflect on their previous investment conditions to facilitate learning and improvement. These processes create changes in agents’ investment strategies and utility functions.

### 3.5. Characteristics of Stock Return Rate in ASM

The agents in the proposed ASM are heterogeneous, exhibit different risk appetites, and adopt various forecasting methods. They are capable of learning and are dynamic and adaptable. The market behaviors of the agents are simulated for 309 periods and the data packets are collected.

**Figure 4:**
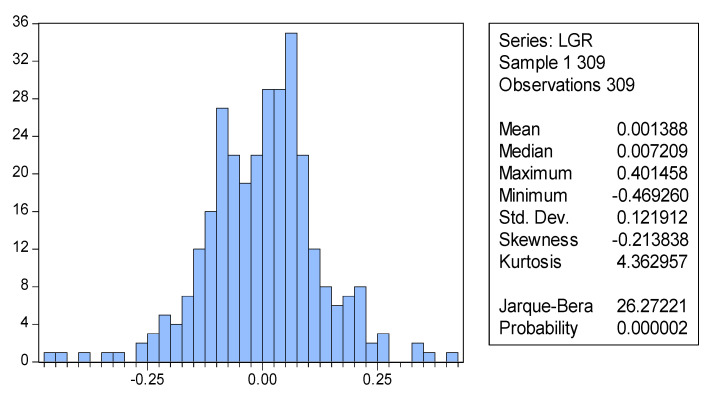
The distribution of the stock log return based on the learning mechanisms.

Figure 4 is a distribution chart of logarithmic rates of return calculated based on the closing prices of the stock market stimulated for 309 periods. The results of a Jarque–Bera (J–B) test indicate that the p values are extremely close to 0. Thus, the hypothesis that the logarithmic rates of stock return follow normal distribution is rejected. The figure shows that S = −0.213838 and K = 4.362957, suggesting that the distribution of the rate of asset trends in the simulation results presented increased skewness and kurtosis.

**Figure 5:**
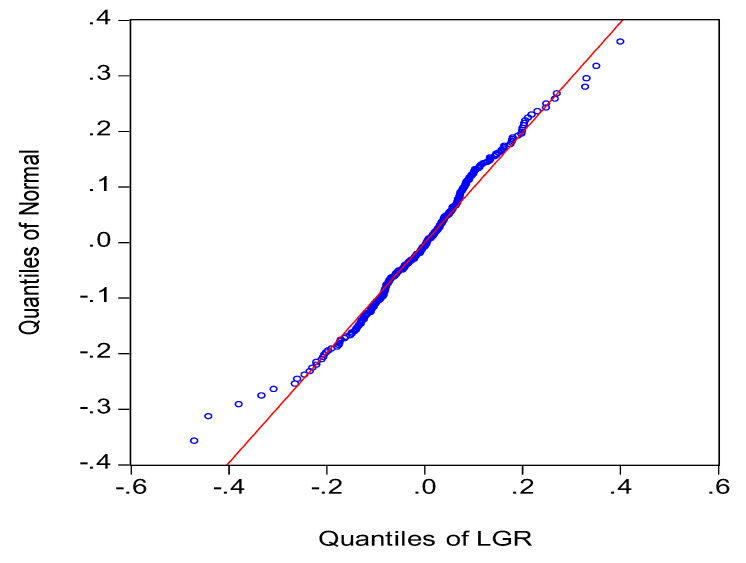
The quantiles of the logarithmic rates of stock return based on the learning mechanisms.

The quartile diagram illustrated in Figure 5 shows that the distribution conditions of rate of return largely conform to a normal distribution, with slight skewness at the extremes, reflecting “fat-tailed” distribution and “dispersed left side” characteristics.

The stock log return statistics indicate that the rate of return in the stock market conforms to a “peaking,” “fat-tail,” and “left-trending” normal distribution. Subsequently, the left fat-tail is more apparent than the right. This explains why small probability events, such as stock market crashes and financial crises, are more likely to occur than previously considered. The results obtained in the present study are consistent with a number of previous empirical studies, verifying that the ASM developed in the present study adequately reflects real-world conditions.

### 3.6. Effects of Agents’ Learning Mechanism on Rate of Return

Several experiments are designed and compared with the results obtained in Section 3.5 to determine the influences that agents’ learning ability and learning frequency have on the market.

**Figure 6:**
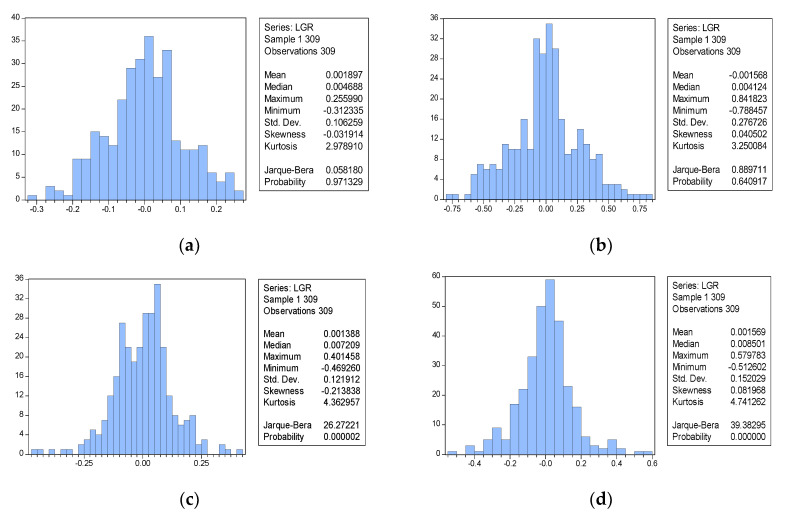
Distribution of stock log return under different learning frequencies. (**a**) No Learning Mechanism; (**b**) t = 1; (**c**) t = 20; (**d**) t = 30.

**Figure 7:**
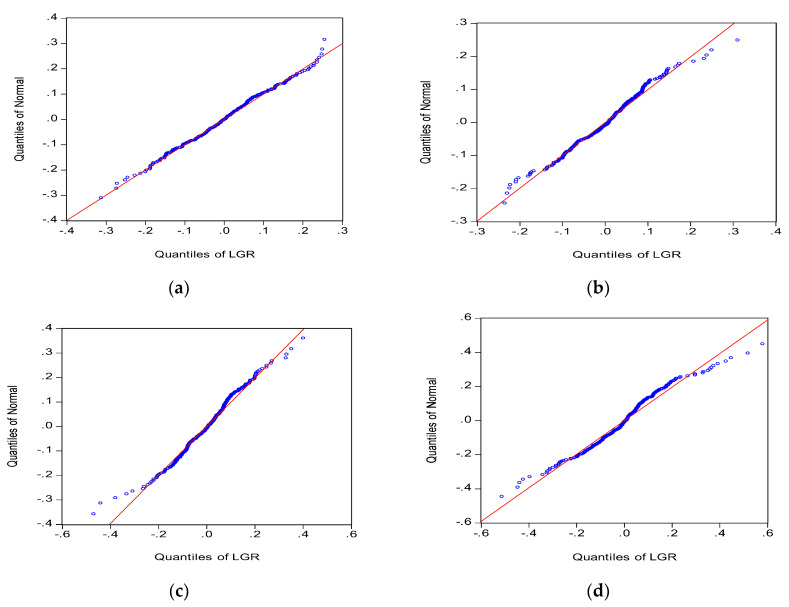
Quantile characteristics of the market rate of return under different learning frequencies. (**a**) No Learning Mechanism; (**b**) t = 1; (**c**) t = 20; (**d**) t = 30.

An experimental group and three control groups are established. The experimental group comprises the agents categorized in Section 3.5 and a learning cycle of 20 days. The first control group contains the same agents with no learning ability. The second control group contains the same agents with a learning cycle of 30 days. The third group contains the same agents with a learning cycle of 1 day. The groups are examined to determine the distribution characteristics of the rate of stock return in different situations. To enhance the comparability of the simulation results of the four groups, we adopt the same random seed in simulating four conditions to obtain the distribution and quantile diagrams of the stock log return for the four conditions (Figure 6 and Figure 7).

The results of the four experiments show that the skewness values for the agents in Group 1 and Group 3 are −0.03 and 0.04, respectively, which are relatively close to 0, and their kurtosis values are 2.98 and 3.25, respectively, which are relatively close to 3. Therefore, the market rate of return for Group 1 and Group 3 approximates a normal distribution. Subsequently, the skewness and kurtosis of market rate of return increase concurrently with an increase in learning cycle. The experimental group achieves S = −0.21 and K = 4.36. Group 2 achieves S = 0.08 and K = 4.74. Moreover, the *p*-values for the J-B statistics of the experimental group and Group 2 are close to 0, suggesting that the original hypothesis is rejected (Table 2).

The Figure 7 illustrates different tail characteristics in the four groups. Group 1 achieves a near normal distribution. The remaining groups show scattering at the extremes, suggesting that the distribution possesses fat-tail characteristics. Subsequently, the fat-tailed distribution is different at the two extremes.

When agents in the market are incapable of learning and adopt a fixed investment strategy, the market outcomes can be determined by summing the linear decision-making behaviors of each agent. Therefore, the rate of return in this type of market exhibits a normal distribution. The results of the groups indicate that the distribution of the market rate of return is more normalized during no or high agent learning conditions. By comparison, the rate of return in markets with longer learning frequencies follows a non-normal distribution and exhibits kurtosis, skewness, and fat-tailed distribution characteristics. Subsequently, non-normal distribution becomes increasingly apparent as the learning interval increases, but only within a specific range. By contrast, when agents are able to engage fully in learning regardless of cost (without considering frictional costs), they continuously review their investment performance and observe and learn the trading methods of others. The overall market is efficient and the rate of return follows a logarithmic normal distribution. When agents in the market are neither capable of learning nor adopt a fixed strategy, they exhibit a nonuniform change in strategic learning. This leads to different agents choosing new strategies at different periods. Agents’ different learning methods and frequencies and their inconsistencies in changing strategies consequently lead to the nonnormalized rate of return in the overall market.

## 4. The Power Law Characteristics of Stock Price Jump Intervals

### 4.1. The Empirical Studies on Several Markets

In paper [1] and [2], the jump diffusion model is as follows:

(13) $$\frac{dS(t)}{S(t-)}=\mu dt+\sigma dW(t)+d\left(\sum_{i=1}^{N(t)}\left(V_{i}-1\right)\right)$$

where S(t) is the asset price, W(t) is standard Brownian motion, N(t) is the Poisson process with rate λ, and $\left\{V_{i}\right\}$ is a sequence of independent identically distributed non-negative random variables. The actual effect will be compared when N(t) is Poisson process and renewal process with power-law nature respectively. Candidate models are set as follows:

Model (1): Forkker–Planck: $f(x)=Ax^{-\alpha }e^{-\frac{\beta }{x}},\alpha >0,\beta >0$;
Model (2): exponential distribution: $f(x)\sim \lambda e^{-\lambda x},\lambda >0$

where x is the interval between two adjacent jumps, f(x) is the probability that jump happens after x.

Barndorff-Nielsen and Shephard (2004, 2006) put forward the bipower variation method (hereafter BPV) to detect jumps [41,42]. The jump detecting method is as follows: Dividing a day-trading-time into M intervals, there have M incomes. In a day, M+1 datum will be observed as $p_{i}(t-1),\cdots p_{i}(t-1+1/M)$. $p_{t}$ is the Log-price of a single asset, *p* is assumed to be Brownian semi-martingale plus jump. The *j*th payoff in a trading day is noted as $r_{i,t,j}=p(t-1+\frac{j}{M})-p(t-1+\frac{j-1}{M})$. Realized volatility is defined by $RV_{i,t}=\sum_{j=1}^{M}r_{{}_{i,t,j}}^{2}$. The bipower variation is defined as $BV_{i,t}=\sum_{j=1}^{M}\mu^{-2}(\frac{M}{M-1})\sum_{j=2}^{M}\left|r_{i,t,j=1}\right|\left|r_{i,t,j}\right|$, where $\mu =\sqrt{2/\pi }≃0.7979$.The feasible jump test statistic is defined by $ZJ_{t}=\frac{RJ_{i,t}}{\sqrt{0.609\frac{1}{M}\max(1,\frac{TP_{i,t}}{BV_{i,t}^{2}})}}$, where $RJ_{i,t}=\frac{RV_{i,t}-BV_{i,t}}{RV_{i,t}}$ is called the ratio jump; $TP_{i,t}=1.7432M\sum_{j=3}^{M}{\left|r_{i,t,j-2}r_{i,t,j-1}r_{i,t,j}\right|}^{4/3}$. $ZJ_{t}\overset{L}{\to }N(0,1)$. If the ratio jump statistic is smaller than its corresponding 95% critical value, then we reject the hypothesis of there being no jump in this unit of time.

The jump interval power law on the Shanghai Stock Exchange Composite Index (SHCI) and S&P 500 Index are severally studied. The data sampling interval is set to 2010.1.4–2016.11.30. There are 1675 SHCI samples, and S&P 500 Index 1730 samples. According to the BPV method, the ratio of jump statistics in unilateral confidence level is 95%, SHCI is detected 81 jumps, S&P 500 Index is detected 58 jumps. We set unit time as 5 days; each unit of time has five observations.

**Figure 8:**
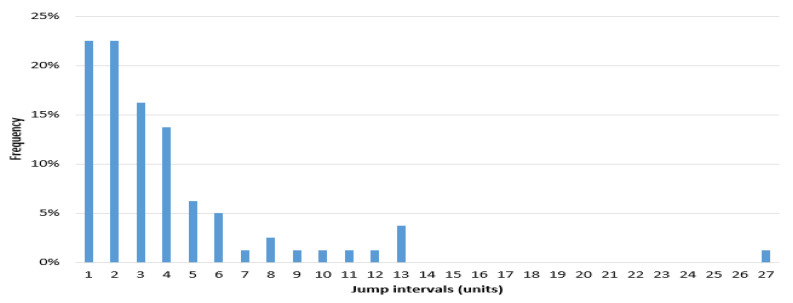
Frequency histogram of Shanghai Stock Exchange Composite Index (SHCI)’s jump intervals.

**Figure 9:**
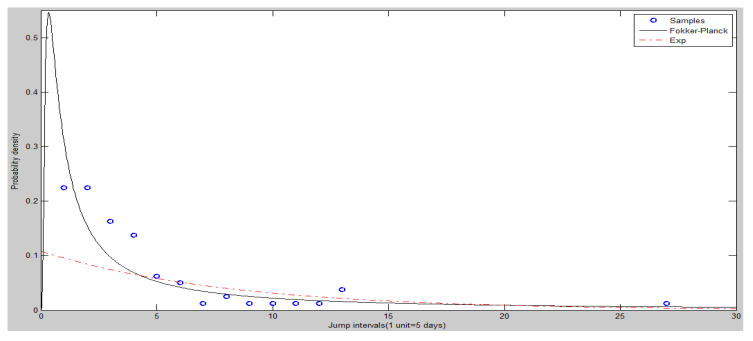
The heads (left hand side) of two amended fit distributions of SHCI.

**Figure 10:**
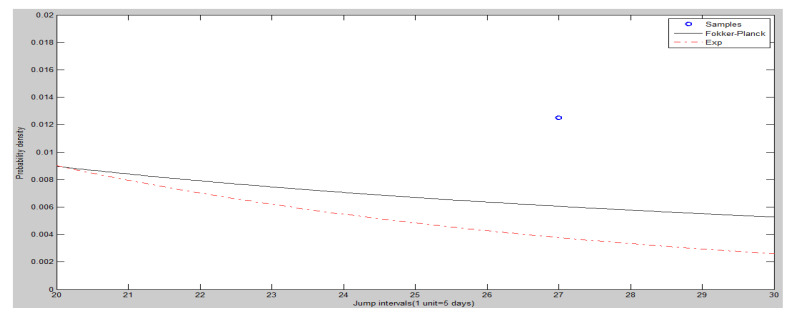
The tails (right hand side) of two amended fit distributions of SHCI.

The frequency histogram of SHCI’s jump intervals is shown in Figure 8. The Figure 9 and Figure 10 show the heads and tails of two amended fit distributions. It is shown that the Fokker–Planck distribution has a better fitting effect in Figure 9 and Figure 10, which show the heads and tails of two amended fit distributions. The Fokker–Planck distribution with the features of sharp kurtosis and fat tail is more matched with human behavior dynamics than the exponential distribution. A higher probability of the tail is given by the Fokker–Planck distribution rather than the exponential distribution.

Table Table 4 shows the fit goodness of model (1) and (2). A comparison of the adjusted R square is performed to judge the models’ goodness of fit. The sum of squared errors (hereafter SSE) and root mean square error (RMSE) will also be compared. Shown in Table 3, it is obvious that the Fokker–Planck distribution is better than the exponential distribution. Table 4 shows the coefficients and 95% confidence interval of each fit function.

**Figure A1:**
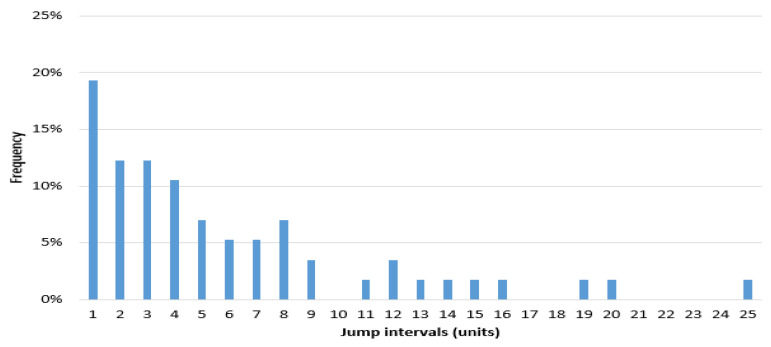
Frequency histogram of S&P 500 Index’s jump intervals.

**Figure A2:**
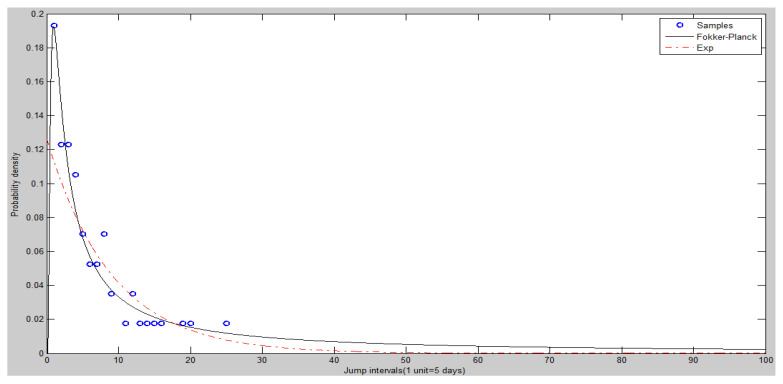
The heads of two amended fit distributions of S&P 500 Index.

**Figure A3:**
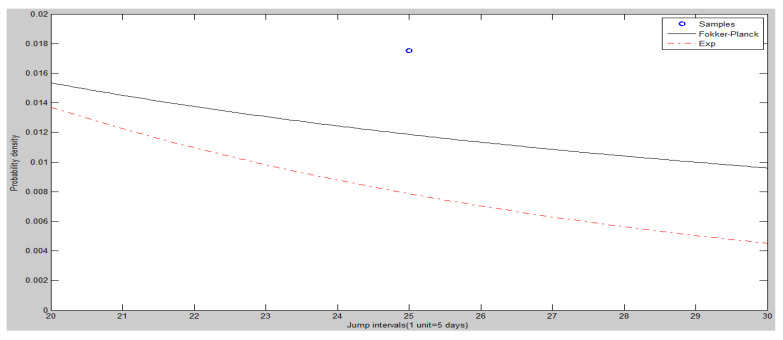
The tails of two amended fit distributions of S&P 500 Index.

The empirical results of S&P 500 Index are shown as Table A5 and Table A6 and Figure A1, Figure A2 and Figure A3 in Appendix C. A similar result in the Hang Seng Index (HSI) in Hong Kong had been empirically achieved in [6]. All the empirical results show that there is an obvious power law of stock price jump intervals in any stock market index.

### 4.2. Analysis of Experiments on ASM

The continuous stock prices of 5010 periods are produced in the experiment simulated in our ASM. Using the method described in Section 4.1, the number of jumps per week (*t* = five days) and the frequencies of each jump quantity are calculated. One hundred and two jumps are detected.

**Figure 11:**
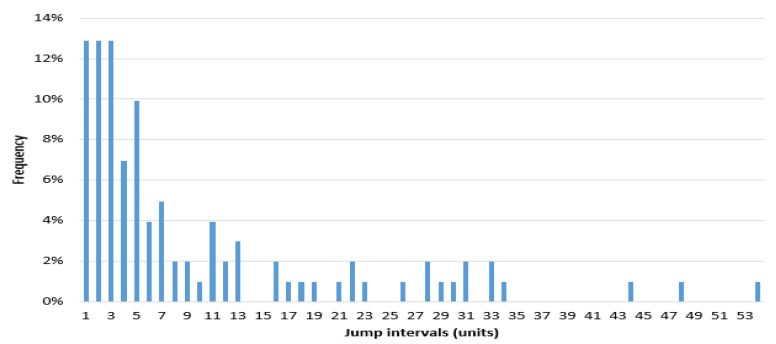
Frequency histogram of experiment (5010)’s jump intervals.

**Figure 12:**
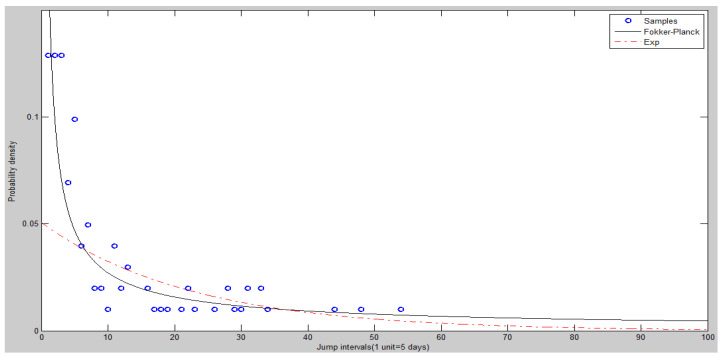
The heads (left hand side) of two amended fit distributions of experiment (5010).

**Figure 13:**
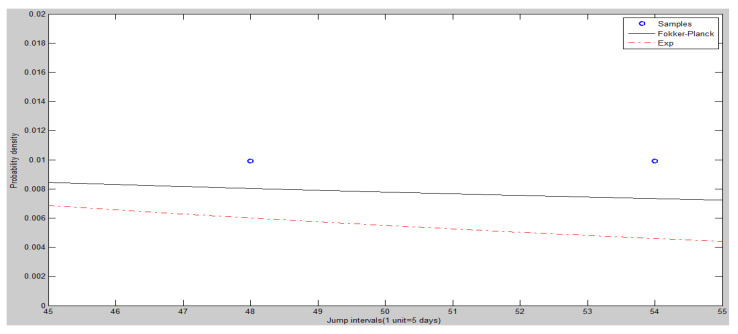
The tails (right hand side) of two amended fit distributions of experiment (5010).

Figure 11 is a frequency histogram of the price jump intervals of the experiment (5010). Figure 12 and Figure 13 show the heads and tails of two amended fit distributions to the 5010 simulation data. Table 5 shows the fit goodness of model (1) and (2) to the experiment (5010). Table 6 shows the coefficients and 95% confidence interval of each fit function. All these results of the experiment show that the ASM can capture the power law characteristics of the stock price jump. The adaptive and heterogeneous agents’ behaviors are the key to drive the power law of the stock price jumps.

In the next study, we examine whether the compliance of changes in stock prices to the power law of jumps is caused by the diversity of agents’ strategies and their learning ability and whether similar results can be obtained when agents’ strategy compositions are changed. For this experiment, two individual control experiment groups are designed. In Experiment Group 1, all agents adopt the trend investment strategy (no learning mechanism). In Experiment Group 2, the design of the agents is similar to that proposed in Section 3.1. To eliminate contingency, each experiment group is repeated 10 times. The ASM is simulated for 3010 periods in each experiment. The method proposed in Section 4.1 is adopted to perform a regression analysis on the data packets obtained from the simulations. The results of Fokker–Planck distribution are tabulated in Table 7.

The result shows that the R-squared values of the two experiment groups are relatively high, suggesting that the model achieves a favorable overall goodness-of-fit, particularly for Experiment Group 2. Although the R-squared values of Experiment Group 1 are lower than those of Experiment Group 2, the results of the first experiments also indicate that the model achieves a favorable goodness-of-fit. It can be inferred that the heterogeneous agents’ different risk appetites and actives are the essential drive mechanism.

The performance of Experiment Group 2 is substantially more favorable than that of Experiment Group 1, suggesting that when the market only comprises agents who adopt the same kind of investment strategy, the power law of the stock price jump interval is weak. By comparison, when agents that adopt diverse strategies are simultaneously present in the market, and they are capable of adjusting their strategies through learning, the changes in stock prices strongly characterize the power law. Paying attention to the mean of the amount of jumps, Experiment Group 2’s is bigger than ExperimentGgroup 1’s, suggesting that the strategic diversity and the agent’s learning promote a jump’s probability.

## 5. Conclusions

In this paper, we study the power law of the stock price jump interval empirically and experimentally. Although the theory assumes the counting process of the jumps is Poisson process, we find that a renewal process with power-law is better suited to the counting process through the empirical study of the Chinese and American stock market. We analyze the production mechanism of the power law by doing experiments in a constructed ASM. 

Unlike previous studies, which only categorize agents using a single attribute, investment styles (strategies) and agents’ basic attributes all are taken into consideration. Agents’ learning ability is also incorporated into the model, and agents’ withdrawal, entry and substitution conditions are considered, creating a dynamically changing market comprising adaptive agents. The results of a preliminary experiment indicate that the proposed ASM accurately reflects the characteristics commonly observed in real markets.

By experiment, it can be determined that agents’ learning ability and learning frequency have influences on the rate of return. Results show that when agents in the market are incapable of learning and when agents are able to learn unconditionally without cost, the market rate of return approximates a normal distribution. The market tends toward non-normal distribution as agents’ learning frequencies reduce, creating a fat-tailed distribution condition with a more dispersed left side. Data packets obtained from simulating the ASM for 5010 periods are incorporated into a regression analysis. Analysis results shows the ASM can capture the power law characteristics of the stock price jumps. 

By comparison with two individual control experiment groups in the ASM, it can be inferred that the diversity and dynamic changes of investment strategies in the market are the more important reasons causing the power law of stock price jumps while the heterogeneous agents’ different risk appetites and actives are the essential drive mechanism. 

We empirically find there are also different characteristics of the power law from different markets. Explaining these differences requires further research.

## Figures and Tables

**Table 1:** Other market parameters.

Parameters	Definition	Value
$\mu_{i,t}$	The expected rate of return of agent *i* in period *t*	$R_{i,t}^{e}$
σ	Standard deviation of daily rate of return	0.01700 *
r	Risk-free rate of return	0.01% **
$p_{0}$	Initial price (opening price in period 0)	10
T	Number of market cycles	5010
α	Risk aversion coefficient during gain	Shown as Table A2
β	Risk aversion coefficient during loss	Shown as Table A3
λ	Loss sensitivity	Shown as Table A4

* Computing method for variance of daily return: first, the standard deviation of daily return is calculated for Shanghai Composite Index 1991–2016 with each year regarded as a period; the mean is calculated for daily standard deviation for each period. ** The calculation method for risk-free rate of return: one-year time deposits are treated as the risk-free rate of return; the average daily return is calculated while continuously compounded.

**Table 2:** Distribution of the market rate of return in different learning frequencies.

	Skewness	Kurtosisi	Jarque–Bera	P
**No learning mechanism**	−0.03	2.98	0.06	0.97
***t* = 30**	0.08	4.74	39.38	0.00
***t* = 20**	−0.21	4.36	26.27	0.00
***t* = 1**	0.04	3.25	0.89	0.64

**Table 3:** The fit distributions’ goodness of SHCI (unit of time: 5 days).

Candidate Fit Distribution	R-Square	Adjusted R Square	F Statistic	*p*-Value	SSE	RMSE
**Fokker–Planck**	0.7507	0.7410	16.5650	0.0005	4.4886	0.5876
**Exponential distribution**	0.4744	0.4609	10.8290	0.0065	9.4655	0.8533

**Table 4:** Coefficients’ values of the fit distributions of SHCI (unit of time: 5 days).

Fit Distribution	Coefficient	Value	95% Confidence Interval
**Fokker–Planck**	lnA	−0.7257	(−3.4551, 2.0037)
	α	1.3242	(0.2678, 2.3807)
	β	0.4439	(−3.0849, 3.9728)
**Exponential distribution**	lna	−2.2255	(−3.0905, −1.3606)
	b	0.1242	(0.0420, 0.2065)

**Table 5:** The fit distributions’ goodness of experiment (5010) (unit of time: 5 days).

Fit Distribution	R-Square	Adjusted R Square	F Statistic	*p*-Value	SSE	RMSE
**Fokker–Planck**	0.7571	0.7497	42.0691	0.0000	5.5205	0.4363
**Exponential distribution**	0.4949	0.4847	27.4318	0.0000	11.4784	0.6291

**Table 6:** Coefficients’ values of the fit distributions of experiment (5010) (unit of time: 5 days).

Fit Distribution	Coefficient	Value	95% Confidence Interval
**Fokker–Planck**	lnA	−1.8497	(−2.9274, −0.7721)
	α	0.7692	(0.4346, 1.1038)
	β	−0.1464	(−1.8067, 1.5139)
**Exponential distribution**	lna	−2.9846	(−3.3991, −2.5702)
	b	0.0444	(0.0271, 0.0618)

**Table 7:** The fit distributions’ goodness of Experiment group 1, 2.

	Experiment Group 1			Experiment Group 2		
Experiment #	R-squared	Adjusted R Square	Jump’s #	R-squared	Adjusted R Square	Jump’s #
**1**	0.3897	0.3064	26	0.8093	0.8001	66
**2**	0.4567	0.4179	31	0.7805	0.7721	55
**3**	0.4017	0.4017	27	0.8191	0.8191	58
**4**	0.7178	0.7178	37	0.7987	0.7987	45
**5**	0.6899	0.6899	32	0.7637	0.7637	49
**6**	0.5625	0.5625	27	0.7409	0.7409	62
**7**	0.3674	0.3674	36	0.7867	0.7867	55
**8**	0.6611	0.6611	29	0.8275	0.8275	46
**9**	0.6581	0.6581	32	0.8812	0.8812	49
**10**	0.7012	0.7012	38	0.8021	0.8021	58
**Mean value**	0.5606	0.5484	31.5	0.8010	0.7992	54.3
**SD**	0.1428	0.1588	4.35	0.0382	0.0387	6.96

## Appendix A

$$\begin{array}{l} \int_{0}^{+\infty }u^{+}(R_{i,t})f(u^{+}(R_{i,t}))du^{+}(R_{i,t})\\ =\int_{0}^{+\infty }u^{+}*{\left[\frac{1}{\sqrt{2\pi }\sigma }e^{-\frac{{\left(\frac{w_{i,t-1}{\left(u^{+}\right)}^{1/\alpha }}{\xi_{i,t}}-\mu \right)}^{2}}{2\sigma^{2}}}*\frac{w_{i,t-1}}{\alpha \xi_{i,t}}{\left(u^{+}\right)}^{\frac{1}{\alpha -1}}\right]}^{}du^{+}\\ =\int_{0}^{+\infty }\xi_{i,t}{}^{\alpha }y^{\alpha }w_{i,t-1}{}^{-\alpha }f(y)\alpha^{-1}\xi_{i,t}{}^{-1}\xi_{i,t}{}^{1-\alpha }y^{1-\alpha }w_{i,t-1}{}^{\alpha -1+1}\xi_{i,t}{}^{\alpha }w_{i,t-1}{}^{-\alpha }\alpha y^{\alpha -1}dy\\ =\int_{0}^{+\infty }w_{i,t-1}{}^{-\alpha }\xi_{i,t}{}^{\alpha }y^{\alpha }f(y)dy\\ \int_{-\infty }^{0}u^{-}(R_{i,t})f(u^{-}(R_{i,t}))du^{-}(R_{i,t})\\ =\int_{-\infty }^{0}u^{-}*{\left[\frac{1}{\sqrt{2\pi }\sigma }e^{-\frac{{\left(\frac{-w_{i,t-1}{\left(-u^{-}\right)}^{1/\beta }}{\xi_{i,t}}-\mu \right)}^{2}}{2\sigma^{2}}}*\frac{w_{i,t-1}}{\beta \lambda^{1/\beta }\xi_{i,t}}{\left(-u^{-}\right)}^{\frac{1-\beta }{\beta }}\right]}^{}du^{-}\\ =\int_{-\infty }^{0}-\lambda \xi_{i,t}{}^{\beta }{\left(-y\right)}^{\beta }w_{i,t-1}{}^{-\beta }f(y)\beta^{-1}\lambda^{\frac{1-\beta }{\beta }}\xi_{i,t}{}^{1-\beta }{\left(-y\right)}^{1-\beta }w_{i,t-1}{}^{\beta -1+1}\lambda^{-\frac{1}{\beta }}\xi_{i,t}{}^{-1}*\lambda \xi^{\beta }w_{i,t-1}{}^{-\beta }\beta {\left(-y\right)}^{\beta -1}dy\\ =-\int_{-\infty }^{0}\lambda \xi_{i,t}{}^{\beta }w_{i,t-1}{}^{-\beta }{\left(-y\right)}^{\beta }f(y)dy\\ V_{i,t}(R_{i,t})=\int_{0}^{+\infty }u^{+}(R_{i,t})f(u^{+}(R_{i,t}))du^{+}(R_{i,t})+\int_{-\infty }^{0}u^{-}(R_{i,t})f(u^{-}(R_{i,t}))du^{-}(R_{i,t}) \end{array}$$

## Appendix B

Table A1 shows the agents’ category and the number of agents in each category. Table A2, Table A3 and Table A4 contain the corresponding α, β and λ parameter values of each type of agent in Table A1, respectively. For example, the corresponding parameters for agents in A_1_ are α = 0.5, β = 1, and λ = 1 and those for agents in D_2_ are α = 0.25, β = 0.3, and λ = 1.5. Agents in I_1_, I_2_, and I_3_ are institute agents. Let α = β, $\frac{\int_{0}^{+\infty }y_{i,t}{}^{\alpha }f(y_{i,t})dy_{i,t}}{\int_{-\infty }^{0}{\left(-y_{i,t}\right)}^{\beta }f(y_{i,t})dy_{i,t}}=\lambda$.

**Table A1:** Agent categorization conditions.

**A**_1_(4).	B_1_(4)	C_1_(4)	D_1_(4)	E_1_(4)	F_1_(4)	G_1_(4)	H_1_(4)	I_1_(4)
A_2_(4)	B_2_(4)	C_2_(4)	D_2_(4)	E_2_(4)	F_2_(4)	G_2_(4)	H_2_(4)	I_2_(4)
A_3_(4)	B_3_(4)	C_3_(4)	D_3_(4)	E_3_(4)	F_3_(4)	G_3_(4)	H_3_(4)	I_3_(4)

Note: The number in parenthesis represents the number of agents. Please refer to the study for details on agent categorization.

**Table A2:** α parameter list.

0.5	0.375	0.375	0.25	0.25	0.125	0.125	0.001	——
0.5	0.375	0.375	0.25	0.25	0.125	0.125	0.001	——
0.5	0.375	0.375	0.25	0.25	0.125	0.125	0.001	——

**Table A3:** β parameter list.

1	0.75	0.5	0.3	0.75	0.5	0.25	0.05	——
1	0.75	0.5	0.3	0.75	0.5	0.25	0.05	——
1	0.75	0.5	0.3	0.75	0.5	0.25	0.05	——

**Table A4:** λ parameter list.

1	2	2.5	1.5	2.5	1.5	2	3	——
1	2	2.5	1.5	2.5	1.5	2	3	——
1	2	2.5	1.5	2.5	1.5	2	3	——

## Appendix C

The data sampling interval of S&P 500 Index is set to 2010.1.4–2016.11.30. There are 1730 samples. According to the BPV method, the ratio of jump statistics in unilateral confidence level is 95%, while 58 jumps are detected in the S&P 500 Index.

**Table A5:** The fit distributions’ goodness of S&P 500 Index (Unit of time: 5 days).

Candidate Fit Distribution	R-Square	Adjusted R Square	F Statistic	*p*-Value	SSE
**Fokker–Planck**	0.9061	72.3368	0.0000	1.1434	0.2593
**Exponential distribution**	0.7870	59.1215	0.0000	2.5924	0.3905

**Table A6:** Coefficients’ values of the fit distributions of S&P 500 Index.

Fit Distribution	Coefficient	Value	95% Confidence Interval
**Fokker–Planck**	lnA	−0.5194	(−1.5622, 0.5233)
	α	1.2021	(0.8238, 1.5805)
	β	1.1314	(−0.2613, 2.5240)
**Exponential distribution**	lna	−2.0685	(−2.4492, −1.6878)
	b	0.1111	(0.0804, 0.1417)

## References

[1] Merton R.C. (1976). Option pricing when underlying stock returns are discontinuous. J. Financ. Econ..

[2] Kou S.G. (2002). A jump diffusion model for option pricing. Manag. Sci..

[3] Hernando A., Plastino A. (2012). Thermodynamics of urban population flows. Phys. Rev. E.

[4] Zambrano E., Hernando A., Bariviera F.A., Hernando R., Plastino A. (2015). Thermodynamics of firms’ growth. J. R. Soc. Interface.

[5] Albert R., Barabási A.-L. (2005). Statistical mechanics of complex networks. Rev. Mod. Phys..

[6] Hernando A., Hernando R., Plastino A., Plastino A.R. (2012). The workings of the maximum entropy principle in collective human behavior. J. R. Soc. Interface.

[7] Eliazar I. (2015). Power-law and exponential rank distributions: A panoramic Gibbsian perspective. Ann. Phys..

[8] Barabási A.L. (2005). The origin of bursts and heavy tails in human dynamics. Nature.

[9] Vazquez A. (2007). Impact of memory on human dynamics. Phys. A Stat. Mech. Appl..

[10] Vazquez A., Rácz B., Lukács A.L. (2007). Impact of Non-Poissonian Activity Patterns on Spreading Processes. Phys. Rev. Lett..

[11] Cao H., Li Y., He Z. (2011). Stock pricing model: Jump diffusion model of power law. J. Manag. Sci. China.

[12] Cao H., Li Y., He H., He Z. (2016). Jump Intervals of Stock Price Have Power-Law Distribution: An Empirical Study. J. Math. Financ..

[13] Farmer J., Foley D. (2009). The economy needs agent-based modelling. Nature.

[14] De bondt W.F.M., Thaler R. (1985). Does the Stock Market Overreact. J. Financ..

[15] Shefrin H., Statman M. (1985). The Disposition to Sell Winners Too Early and Ride Losers Too Long: Theory & Evidence. J. Financ..

[16] Odean T. (1998). Are investors reluctant to realize their losses?. J. Financ..

[17] Thaler R.H. (1987). Amomalies: The January effect. J. Econ. Perspect..

[18] Tversky A., Kahneman D. (1992). Advances in Prospect Theory: Cumulative representation of uncertainty. J. Risk Uncertain..

[19] Li X.D., Wang J.I., Fu H. (2002). Investigations on the Transaction Behaviors of Chinese Individual Securities Investors. Econ. Res. J..

[20] Yang C.P., Wu C.F., Chen M. (2005). Behavioral Finance: Understanding Risk and Expected Return. Chin. J. Manag. Sci..

[21] Zhang W. (2010). Computational Experiment Finance.

[22] Holland J.H. (1992). Adaption in Natural and Artificial Systems: An Introductory Analysis with Application to Biology. Control and Artificial Intelligence.

[23] Kirman A., Gilles T. (2001). Microeconomic models for long-memory in the volatility of financial time series. Stud. Nonlinear Dyn. Econ..

[24] Gao B.J., Dai H., Xuan H.Y. (2005). An Agent-based Stock Market Simulation Model. Syst. Eng. Theory Methodol. Appl..

[25] Zhang Y.J., Zhang W., Xiong X. (2010). Strategies and Investment Returns: Agent-Based Computational Finance Perspective. J. Manag. Sci. China.

[26] Zhang H.F., Zou G.F. (2011). The Comparison Analysis of Different Decision Preferences Based on ACF Method. J. Guangdong Univ. Financ..

[27] Kyle A.S. (1989). Informed Speculation with Imperfect Competition. Rev. Econo. Stud..

[28] Kyle A.S. (1984). Market Structure, Information, Futures Markets and Price Formation. International Agricultural Trade: Readingsin Price Formation, Market Structure, and PriceInstability.

[29] Klugera B.D., McBrideb M.E. (2011). Intraday trading patterns in an intelligent autonomous agent-based stock market. J. Econ. Behav. Organ..

[30] Foucault T., Kadan O., Kandel E. (2005). Limit Order Book as a Market for Liquidity. Rev. Financ. Stud..

[31] Ma Z.X., Zhang W., Xiong X., Zhang Y.J. (2011). Research on the Impact of Composition of Traders on Market Based on Agent-based Computational Finance. J. Econ. Issues.

[32] Chakrabarti R., Roll R. (1999). Learning from others, reacting, and market quality. J. Financ. Mark..

[33] Hu J., Weliman M.P. (2003). Nash Q-Learning for General-Sum Stochastic Games. J. Mach. Learn. Res..

[34] Chen S.-H., Ye C.-H. (2001). Evolving trader and the business school with genetic Programming: A new architecture of the agent-based artificial stock market. J. Econ. Dyn. Control.

[35] Kendall G., Su Y. The Co-evolution of Trading Strategies in A Multi-agent Based Simulated Stock Market Through the Integration of Individual Learning and Social Learning. Proceedings of the 2003 International Conference on Machine Learning and Applications-ICMLA 2003.

[36] Iettau M. (1997). Explaining the facts with adaptive agents: The case of mutual fund flows. J. Econ. Dyn. Control.

[37] Cincotti S., Focardi S.M., Marchesi M., Raberto M. (2003). Who wins? Study of long-run trader survival in an articial stock market. Phys. A Stat. Mech. Appl..

[38] Brock W.A., Hommes C.H. (1997). A rational route to randomness. Econometrica.

[39] Bernard C., Ghossoub M. (2010). Static portfolio choice under Cumulative Prospect Theory. Math. Financ. Econ..

[40] Zhou H., Jiang J., Zeng W. An agent-based finance market model with the continuous double auction mechanism. Proceedings of the 2010 Second WRI Global Congress on Intelligent Systems.

[41] Barndorff-Nielsen O.E., Shephard N. (2004). Power and bipower variation with stochastic volatility and jumps. J. Financ. Econ..

[42] Barndorff-Nielsen O.E., Shephard N. (2006). Econometrics of testing for jumps in financial economics using bipower variation. J. Financ. Econ..

